# Paediatric enteral feeding at home: an analysis of patient safety incidents

**DOI:** 10.1136/archdischild-2019-317090

**Published:** 2019-06-14

**Authors:** Bethan Page, Rasanat Nawaz, Sarah Haden, Charles Vincent, Alex C H Lee

**Affiliations:** 1 Department of Experimental Psychology, University of Oxford, Oxford, UK; 2 Oxford Academic Health Science Network, Oxford, UK; 3 John Radcliffe Hospital, Oxford, Oxfordshire, UK

**Keywords:** gastroenterology, comm child health, health services research

## Abstract

**Aims:**

To describe the nature and causes of patient safety incidents relating to care at home for children with enteral feeding devices.

**Methods:**

We analysed incident data relating to paediatric nasogastric, gastrostomy or jejunostomy feeding at home from England and Wales’ National Reporting and Learning System between August 2012 and July 2017. Manual screening by two authors identified 274 incidents which met the inclusion criteria. Each report was descriptively analysed to identify the problems in the delivery of care, the contributory factors and the patient outcome.

**Results:**

The most common problems in care related to equipment and devices (n=98, 28%), procedures and treatments (n=86, 24%), information, training and support needs of families (n=54, 15%), feeds (n=52, 15%) and discharge from hospital (n=31, 9%). There was a clearly stated harm to the child in 52 incidents (19%). Contributory factors included staff/service availability, communication between services and the circumstances of the family carer.

**Conclusions:**

There are increasing numbers of children who require specialist medical care at home, yet little is known about safety in this context. This study identifies a range of safety concerns relating to enteral feeding which need further investigation and action. Priorities for improvement are handovers between hospital and community services, the training of family carers, the provision and expertise of services in the community, and the availability and reliability of equipment. Incident reports capture a tiny subset of the total number of adverse events occurring, meaning the scale of problems will be greater than the numbers suggest.

**What is already known?**

- There are increasing numbers of children who require specialist medical care at home. Most of this care is provided by parents.
- While there are advantages of care at home, little is known about the safety of enteral feeding at home.

**What this study adds?**

- This study identifies a range of safety problems occurring with enteral feeding at home, many of which can remain hidden from paediatric services.
- If children with complex care needs are to be cared for safely at home, the provision of services to support families at home need improving.
- Priorities include handovers from hospital to community, training for family carers, provision and expertise of services in the community, and availability and reliability of equipment.

## Introduction

Children with complex medical needs are increasingly cared for at home rather than in hospital.^1^ Family members, with the support of nurses and other healthcare professionals, deliver the day-to-day care these children require. Common procedures carried out by parents include enteral feeding, tracheostomy care and administering intravenous medication. While there are clear advantages of care at home, many of the tasks that are now commonplace in the home have significant safety risks that need to be managed and better understood.^2^

Many children with severe chronic illnesses and neurodisability do not have a safe swallow or are unable to meet their nutritional requirements orally through eating and drinking. Feeding tubes and devices are commonly used to support nutritional needs.^3 4^ Home enteral nutrition was first established over 30 years ago.^5 6^ In a report by the British Artificial Nutrition Survey, it was estimated that there were 16 982 children on home enteral nutrition in the UK in 2010, with an increase of 41.5% between 2005 and 2010.^7^ There are several types of enteral feeding, all of which involve inserting a device into the stomach and/or jejunum. Nasogastric (NG) tube feeding is the the most common short-term solution. Surgically placed devices are required in children with longer-term feeding needs, such as a gastrostomy tube, gastrostomy low-profile ‘button’ or jejunostomy (trans-gastric or directly).

There are many benefits to home enteral nutrition for both the child and family, such as shorter hospital stays and reduced risk of malnutrition-related complications.^8 9^ However, there are also risks. Within a month of discharge following gastrostomy surgery, almost 10% of patients visited the emergency department or were readmitted to hospital.^10^ Common complications for gastrostomies include over-granulation, infection or leaking around the stoma site, and broken or blocked gastrostomy tubes.^11–13^ There are also rare but serious risks such as peritonitis following displacement of a gastrostomy device.^14^ For NG feeding, the most notable risk is feeding through a misplaced NG tube into the lung.^15^ NG tubes can be easily pulled out, especially by babies. There is an increased risk of misplacement with frequent tube replacement.^9^ A series of studies observing parents caring for children with NG or gastrostomy tubes highlighted several safety issues, including deterioration in hygiene practices over time and irregular checking of tube position.^16–18^ Tube-related complications are common with enteral feeding,^12 19^ but it is unclear to what extent these could be avoided by improved safety practices.

To date, we have a limited understanding of the risks of providing enteral feeding at home. Analysing incident reports offers a window into the safety of systems, highlighting vulnerabilities and inadequacies, and detecting common problems and rare and serious risks.^20^While there is a large literature analysing incident reports in the hospital environment, there has been very limited exploration of incidents in the community or home setting.^21^ Analysing incidents reported in the community will provide an overview of the types of problems occurring with enteral feeding at home. The aim of this study is to characterise the nature and causes of patient safety incidents involving children with feeding devices at home and to identify priorities for improvement.

## Methods

### Data source

The data source for this study is the National Reporting and Learning System (NRLS). This is a national repository of anonymised patient safety incident reports from National Health Service (NHS) organisations across England and Wales.^22^ Individuals, organisations and NHS Trusts can voluntarily submit reports to the national repository. They are encouraged to report any ‘patient safety incidents’, defined as ‘any unintended or unexpected incident that could have or did lead to harm for one or more patients receiving NHS-funded healthcare’.^22^ They contain categorical information which includes patient demographics, level of harm and location and date of the incident. There are also open text boxes for information about what happened and why it happened. More information about the NRLS data is available on their website.^22^

### Sample selection

A sample of incidents relating to gastrostomy, jejunostomy and NG feeding in paediatrics were requested from NRLS to include incidents reported between 1st August 2012 and 31st July 2017. The following free text search terms were used to identify the incidents: Gast* button, G-button, mickey button, enteral feed, NGT, NG tube, NG feed, naso-gastric feed, naso-gastric tube, jejunostomy feed, jejunostomy tube, jejunal feed, jejunal tube and gastrostomy. A total of 9327 incidents were received from NRLS.

**Figure 1:**
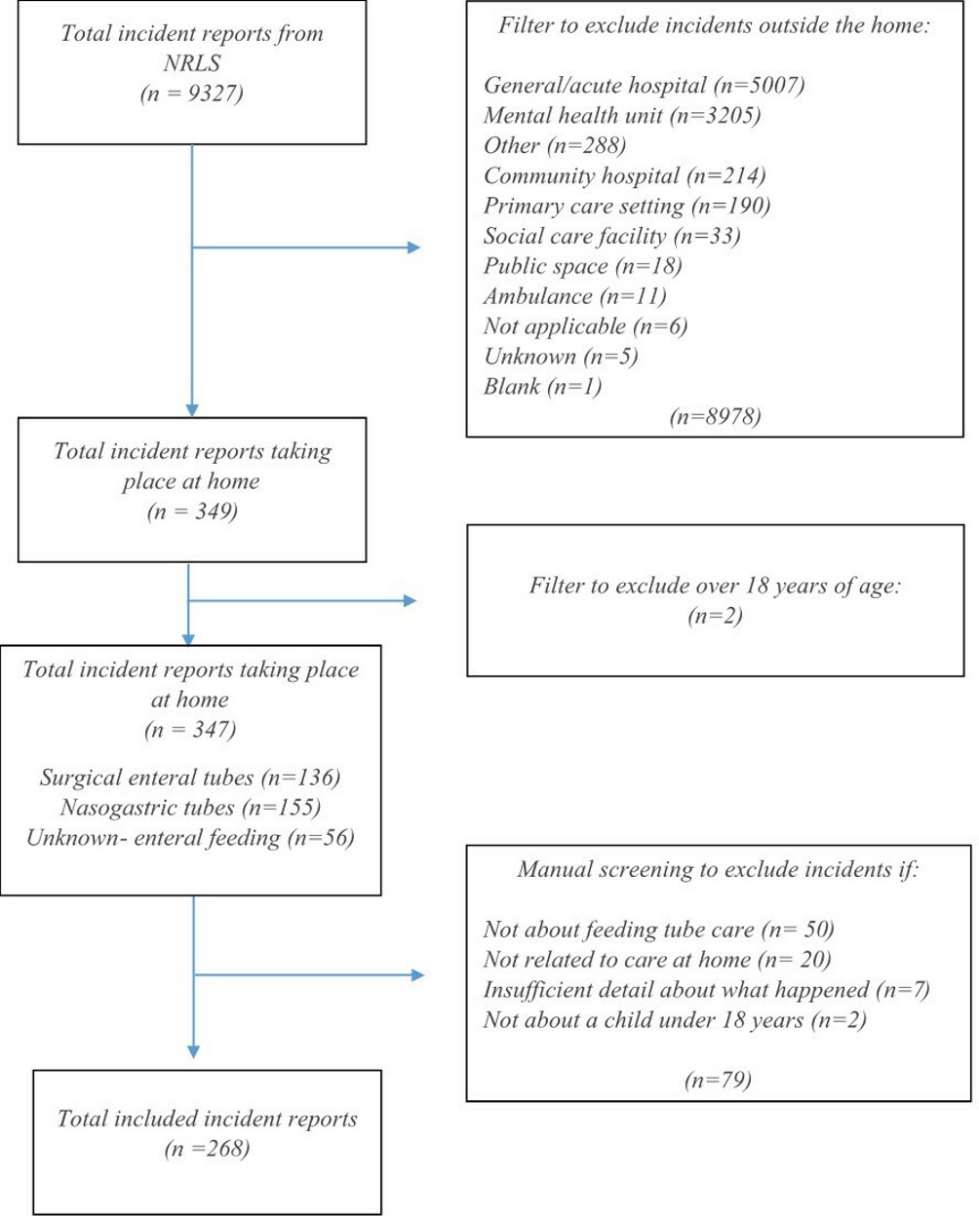
Flow diagram showing the steps taken to identify the final sample of incidents for inclusion.

The incidents were first filtered by reported incident location to identify reports occuring in the home and then by age to remove incidents involving patients over 18 years. These 349 incidents were manually reviewed to exclude incidents without a clear description, not relating to enteral feeding, not relating to home care and any remaining reports involving adults. This produced a final sample of 268 incidents for analysis. Figure 1 shows the flow diagram illustrating the steps taken to identify the sample.

### Analysis

The selected incidents were imported into NVivo, V.12. The free text descriptions were coded to identify the problems in care, any stated contributory factors and patient outcomes. An adapted framework approach was used.^23^ Two authors (BP and RN) familiarised themselves with the data and noted relevant themes. BP and RN coded 10% of the incidents identifying the problems in care, contributory factors and patient outcomes. Initial agreement was over 90%. Discrepancies were resolved through discussion and the two authors then coded half of the remaining incidents each. Incidents which were unclear were discussed with author SH, a community paediatrician, who assisted with the analysis of the clinical circumstances described in these cases. The problems in care, contributory factors and patient outcomes were then grouped into themes and subthemes through an iterative process by adapting existing general incident frameworks to fit the narrow field of enteral feeding in the home.^24–26^ A sample of 10% of the incidents were coded independently by author AL, who has significant clinical expertise in surgical enteral feeding. Agreement was 100% for outcomes and care problems and 95% for contributory factors. Author CV, with expertise in incident analysis, carried out a final check of the frameworks. Example incidents and coding are shown in online supplementary file 1. A more detailed description of the analysis process is available in online supplementary file 2.

10.1136/archdischild-2019-317090.supp1 Supplementary file 1

10.1136/archdischild-2019-317090.supp2 Supplementary file 2

### Ethics

The incidents from the NRLS were anonymised and made available by NHS Improvement through a data sharing agreement with the Oxford Academic Health Science Network (AHSN). This was part of a service improvement project and conducted under the auspices of the Patient Safety Collaborative at Oxford AHSN as part of their regional Specialised Paediatric Care programme.

## Results

### Problems identified in the processes of care

At least one problem in care was identified in each incident, with some incidents having two or three problems. The problems in care fell into nine different categories (see table 1). The most common categories were equipment and devices (n=98, 28%), procedures and treatments (n=86, 24%), information, training and support needs of families (n=54, 15%), feeds (n=52, 15%) and discharge from hospital (n=31, 9%). Incidents occurred across the age span, with 32% occurring in children under 1 year, 26% in children 2–4 years, 27% in 5–11 years and 9% in children 12–17 years (in 6% of incidents the age was unknown).

**Table 1:** Problems in the process of care

Problems in care	N
**Administration and documentation**	**7**
Errors in documentation	4
Documentation not available	3
**Communication**	**7**
Communication failures between staff	3
Inadequate handovers in the community	3
Communication problems between staff and family	1
**Discharge**	**31**
Inadequate or no handover from hospital to community teams	13
Required equipment, medication or feeds not supplied at discharge	9
Other discharge problems	6
Lack of support in the community post-discharge	3
**Equipment and devices**	**98**
Faulty or damaged gastrostomy and jejunostomy devices	25
Faulty or damaged feeding equipment (eg, giving sets, pumps)	24
Faulty or damaged NG tubes	13
Equipment not available	13
Incorrect equipment ordered or delivered	7
Device is leaking or loose	6
Equipment not delivered or delayed	4
Equipment used incorrectly	4
Equipment out of date	2
**Feeds**	**52**
Feed not given on time	12
Incorrect feed or feeding regime given	12
Incorrect feed ordered or delivered	9
Feed given through incorrect port	8
Feed not delivered or delayed	4
Out of date feed delivered or administered	3
Child left unattended during overnight feeding	3
Feed leaking	1
**Information, training and support needs of families**	**54**
Family carer has not received appropriate training or information	28
Family carer does not follow procedure correctly or goes against advice	16
Family carer given inappropriate advice	5
Lack of support for family in the community	3
Family carer given conflicting information	2
**Medications**	**16**
Medication administered through incorrect port	4
Medication inserted into balloon	2
Medication not given	2
Medication or prescription errors	2
Wrong dose given	2
Difficulties obtaining medication	1
Medication blocks tube	1
Medication given at wrong time	1
Wrong medication given	1
**Procedures and treatment**	**86**
Gastrostomy button or jejunostomy device comes out	11
Delays to procedure or no staff available	11
Problems or complications passing NG tube	11
Tube wrapped around neck during overnight feed	6
Wrong length NG tube passed	6
Wrong size button fitted	6
Procedure not followed correctly	6
Problems changing or fitting button	5
Feed, water or medication put down tube without confirming position	5
Complications relating to gastrostomy site	3
Damage from nasal bridle	3
Staff member does not have appropriate training	3
Silver nitrate-related problems	3
NG tube comes out	2
Child pulls out feeding tube during overnight feeding	1
Inappropriate treatment	1
Procedure done on wrong patient	1
**Other**	**2**
Missed appointments or reviews	2

NG, nasogastric.

Table 1 shows the specific problems identified within each of the categories. Faulty and damaged equipment was a very common problem (n=62, 18%). It is unclear to what extent the problems with faulty and damaged equipment are underpinned by poor design or by inappropriate use of equipment. Many of the problems highlighted may relate to inadequate training or knowledge of nurses and paid carers. For example, medications and feeds were administered through the wrong port of a gastrojejunostomy tube in 12 incidents, and in two incidents, medication was wrongly inserted into the balloon part of the gastrostomy button device.

### Outcomes for the child

Table 2 shows the breakdown of outcomes for each incident. There was a clearly stated harm to the child in 52 (19%) incidents, including 17 (6%) incidents which resulted in a hospital admission or accident and emergency visit. Some of the incidents in the potential harm category may have resulted in harm to the child which was not stated by the reporter. In some of the incidents classified as ‘potential harm’, there was a clear potential for harm but no actual harm occurred. An example of this would be where a child was fed through an NG tube despite being unable to obtain aspirate and a suitable pH value.

**Table 2:** Outcomes for the child

Outcomes	N
**Clearly stated harm to child**	**52**
Hospital admission or accident and emergency	17
Skin damage, pain or distress relating to gastrostomy site	12
Diarrhoea, sickness or abdominal pain	7
Feeding tube wrapped around neck	6
Skin damage from nasal bridle	3
Child not fed	2
Pain or distress passing nasogastric tube	2
Child aspirating blood	1
Seizure	1
Hypoglycaemia	1
**Potential for harm (or harm not stated)**	**216**

### Factors contributing to the incidents

There were 97 contributory factors identified in the incidents. In the majority of incidents, no contributory factors were mentioned. Contributory factors fell into five broad categories: organisational factors (32%, n=31), staff factors (21%, n=20), family carer factors (20%, n=19), feeds, equipment and medication factors (12%, n=12) and patient factors (15%, n=15). Table 3 gives definitions and example quotes for each category. Organisational factors such as poor communication between services, lack of service availability and evening and weekend discharges were common. These factors highlight the transition from hospital to home as a particularly risky period and concerns regarding the availability of community services to support families. The circumstances of the family carer, such as the involvement of a secondary family carer (eg, grandparent), ongoing child protection issues or a parent experiencing distress, all affected the provision of care. The training needs of individual staff members was also a common problem and raises questions about the safety of care in the community in some services.

**Table 3:** Types and frequencies of contributory factors with illustrative quotes

Contributory factors	N	Illustrative quotes
**Family carer factors:** features of the family carer or their circumstances that make caring for the child more difficult, or may contribute to problems in care	**19**	**Secondary family carers:** *The child is in the care of the grandma on a Tuesday and Thursday… the Mum had been trained… and was signed her off as competent, but not the grandmother. It seems that she [the Grandma] didn’t want to disturb her daughter so she did it [repassed the NG tube] herself.* **Child protection issues:** *This family have already had a faulty button which has been sent for analysis and reported to have a tear in the balloon in an abnormal place. Cause unknown. This family have previously been subject to child protection issues due to inappropriate use of equipment.*
**Feeds, equipment and medication factors:** factors relating to the design of equipment, feeds and medications which affect the provision of care	**12**	**Similar names of feeds:** *Care required when adding enteral feeds to the ordering system, paying particular attention to the names of feeds as many names are very similar.* **Design of equipment:** *Since the introduction on the new ENFITT connectors provided in the giving set, the connection onto the Mic key button extension is not as secure and therefore the milk feed is leaking.* **Design and durability of equipment:** *The device has an increased risk of leaking if the feed is high in MCT oil or amino acid content which is applicable to the paediatric caseload. They [the feeding company] have advised that some breaks are the results of stress fractures and patients and carers should be encouraged not to turn it too tight.*
**Organisational factors:** features of the way organisations function which affect the provision of care available	**31**	**Staff/service availability:** *Visited family home to pass a routine monthly NGT [nasogastric tube]… Throughout initial discussions on when NGT was last place, mum disclosed that she had called the CCN [community children’s nursing] team on [date]. Unfortunately we were not available to attend and mum advised she did not want to go all the way to the hospital and wait for ages, so she passed it herself.* **Evening and weekend discharges:** *Patient discharged from hospital with Nasogastric tube and feeds, risk assessment not completed properly, risks identified with high PH not highlighted. Parent competency not complete. Community children’s nursing team received referral form post discharge of patient at 17.20pm in evening (outside working hours).* **Poor communication between hospital and community:** *Unable to find any information on a patient’s Trans Gastric Jejunal tube… The nurse needs to know the size of the tubes in order for extension tubes to be sent out to the home on a regular basis. The team have experienced a frequency of similar incidences and generally poor communication with our Teams resulting in families not able to change water in the balloon.*
**Patient factors**: features of a patient that make caring for them more difficult and therefore more prone to error	**15**	**Child distressed during the procedure:** *Child very upset about the procedure [changing a button] screaming and protesting… checked as the fit of the button looked tight… spare 3 cm, in situ two cms… she [Mum] acknowledged she had handed me an old button by mistake because of all the upset.* **Complex medical history:** *Attempting to pass [nasogastric] tube as per protocol to right nostril but unable to advance past 14–15 cm felt may be a blockage, patient limp, loss of colour and unresponsive… Since his discharge home from hospital following his birth the child has had several admissions to hospital. These have been with upper respiratory tract infections and vomiting and had a snotty nose and cold at the time of the incident.*
**Staff factors**: features of individual staff members that may contribute in some way to problems in care	**20**	**Lack of knowledge and preparation:** *When the parent asked her [the nurse] to carry out care of the Freka tube this was something that she had not had time to prepare for in advance and instead of taking time to read through the care plan to clarify what was needed she went ahead and dealt with what she thought was the correct procedure.* **Variability in best practice guidance from consultants:** *The water in the balloon was changed within a week of insertion [of gastrostomy], the best practice guidance is 3 weeks post insertion. No guidance given from the ward on referral or to patient. The practice of inserting pegs or buttons can vary from individual consultants and changing devices. Practitioners unaware of new practice.*

## Discussion

Our analysis of incident reports on enteral feeding at home found a number of safety concerns. Commonly reported problems included faulty and broken equipment and family members not receiving sufficient training or information. Underlying causes included organisational factors and factors relating to staff and family carers. Incident data underestimates the scale of harm so these data represent only a small proportion of the total problems occurring in the community.^27^ Our study highlights a range of safety concerns which require further investigation and action.

Incidents relating to broken or faulty equipment and the availability of equipment were common, and have also been reported in palliative care settings elsewhere.^23^ The cause of faulty or broken equipment is likely to be a mixture of issues in product design and misuse of equipment by parents and staff, and is therefore partly a training problem. It is often impractical for community services to stock all the possible equipment children may need. The number of children one service looks after is relatively small, and the variety of equipment children need can be substantial. Competition between manufacturers helps keep costs lower and creates an incentive for companies to respond to complaints about broken devices and implement design changes. However, this market model can also have unintended consequences such as a multitude of different devices which creates a complex landscape for families and healthcare professionals to navigate.^28^

### Implications for clinical practice and organisation of services

If children with complex medical needs are to be cared for at home, the provision of services to support them needs to be strengthened in a number of respects. First, handovers between hospital and community need improving so that all children are safe in the critical first few weeks at home. Second, family carers need consistently good quality training. Third, sufficient provision of community services is needed with the required expertise to support these families, and fourth, equipment needs to be reliable, with back-up equipment available in the community.

The weeks following discharge from hospital are a high-risk period. We found a number of instances where community teams had not been informed of the child’s discharge, and cases where children were discharged without the required equipment, medication or feeds. It is vital that there is continuity of care between hospital and community services. Pressure to discharge patients due to bed shortages may be increasing the risks. Standardised checklists could help address some of the problems identified at discharge. It can be tempting to think the problems identified in the incidents are mostly related to the transition from hospital and home, but the range of ages of the children involved suggests there are also considerable problems in long-term care.

Inadequate training of family carers was a frequently reported concern. Other studies have found evidence of safety concerns in the practices of some family carers.^16–18^ Many families also worry about making mistakes or feel inadequately prepared.^29 30^ The adequacy of training and information for parents needs to be viewed as a system issue and vital to the safety of care at home.^31^

Underlying a number of the problems identified, is inadequate provision of services in the community to support families. Lack of expertise and availability of services for specialised paediatric care have been identified by others previously, including parents.^32 33^ Our study indicates that there are varying levels of expertise among those who provide care, whether that be parents, Community Children’s Nurses, paid carers or school or respite staff. Established nutritional support teams need sufficient time to train families and other community professionals, to ensure that as much as possible, day-to-day care and minor complications are safely managed at home. There needs to be cross-pollination of expertise across services.

Different surgical/gastroenterological specialists and feeding healthcare professionals use different devices, which creates a complex landscape for parents and community services to navigate. Increased standardisation is needed. Improvements are needed to the reliability of equipment and provision of back-up equipment, or children will miss vital feeds and medications.

Box 1 gives a list of recommendations for action and further investigation. Key recommendations are given for the four major themes identified in this study. If children with complex needs are to be cared for safely at home, services to support families must be strengthened in these four areas.

**Recommendations for action and further investigation**

**Handover between hospital and community**

- There needs to be robust systems to ensure community services are always contacted before the child leaves hospital.
- There should be a standardised checklist used in hospital to ensure families have the appropriate training and equipment, and contact details of their lead community contact, typically a Community Children’s Nurse (CCN).

**Training of family carers**

- Training needs to be carefully planned. The children’s charity Well Child recently produced guidelines for the training of family carers, which provides a useful starting point.^38^
- Simple online training could be made available nationally for parents as part of a training curriculum (eg, to include videos and scenarios). This would also be of benefit to secondary family carers such as grandparents.
- Systems need to be in place to check the competency of family carers.
- There needs to be further clarification between hospital and community services as to who is responsible for delivering what training. Training needs to be delivered by staff with sufficient expertise.
- Through the Oxford Academic Health Science Network (AHSN), we have developed a regional training booklet for gastrostomies which includes competencies. Training begins in the community prior to gastrostomy surgery. The booklet is used by both hospital and community services.

**Provision and expertise of services in the community**

- Multidisciplinary staff training days are needed to ensure cross-pollination of expertise across services. We have run regional training days through the Oxford AHSN attended by CCNs, parents, dieticians, paediatricians, surgeons and hospice staff.
- Expert parents and specialist feeding teams should be involved in staff training.
- Specialist hospital teams need to be available for telephone advice, and patient review as required. Email can be suitable for non-urgent advice. Where possible, lower level problems should be managed in the community.

**Availability and reliability of equipment**

- Further investigation is needed on how to improve the design and durability of equipment. We recommend creating a virtual network of staff working with children with feeding tubes for example, CCNs, dieticians, hospital teams, parents—so that problems with equipment are identified and reported through the network, and then raised with the manufacturers.
- Back up equipment in the community needs to be available for all children in case of delivery problems or faulty equipment. Discussions are needed between hospital and community teams to improve models for the supply of back-up equipment in emergencies.

### Strengths and limitations

Incident reports are excellent tools for learning and for generating improvements to current systems. Patient safety incidents have been extensively studied in the hospital setting, but to date there has been limited research in other care settings.^23 34^ Our study is, to our knowledge, the first to examine incidents relating to the safety of enteral feeding at home. It documents a range of problems that need attention. More broadly, this study begins to examine a new area of research in the field of patient safety: care in the home and the involvement of family members in providing this care.^35^

The limitations of incident reporting have been discussed in detail elsewhere.^36^ Incident reporting generally only detects a small proportion of the total number of adverse events occurring.^27^ Our study therefore cannot comment on the frequency of safety problems with enteral feeding devices at home. We note that the terms ‘PEG’ and ‘PEJ’ were not included in the search terms so it is possible that some relevant incidents were missed. The majority of these reports were written by healthcare professionals and, as a result, our study cannot adequately explore the perspective of parents. Ideally, families should be more involved in incident reporting as they are the primary caregivers.

## Conclusions

This study identifies a range of safety problems occurring with enteral feeding at home, many of which can remain hidden from paediatric services. Incident reports capture a tiny subset of the total number of adverse events occurring, meaning the scale of problems will be much greater than the numbers suggest. Priorities for improvement are checklists to support handovers from hospital to community, ensuring consistently good quality training for family carers, increased cross-pollination of expertise across services and closer working relationships with equipment suppliers to improve the reliability of equipment. Future studies should examine parents’ safety concerns and compare the findings with the themes identified in this study. Previous studies have found that families and patients are able to identify factors which contribute to safety incidents and that these are sometimes different from those identified by healthcare professionals.^37^ The provision of services to support care at home needs to be strengthened.
