# Supplementary material for: Paediatric enteral feeding at home: an analysis of patient safety incidents
Source: Arch Dis Child. 2019 Jun 14;104(12):1174–80. doi: 10.1136/archdischild-2019-317090 (PMC6900243; doi:10.1136/archdischild-2019-317090)
Supplement: Supplementary file 1 [file archdischild-2019-317090supp001.pdf]

Supplementary File 1: Free text examples of incidents with coding

Example 1

Patient discharged from hospital following insertion of gastrostomy tube, community children's nursing team unaware of discharge and parents not sufficiently trained in care of daily management of tube. On home visit assessment skin granuloma forming where flange too tight to skin and daily care not carried out by parents.

Care problem:

i) *Poor communication between hospital and community team at discharge*: CCN team unaware of discharge

ii) *Family carers hasn't received the appropriate training or information:* parents not sufficiently trained

iii) *Family carers hasn't received the appropriate training or information*: parents not carrying out daily management of tube

Contributory factor: None stated

Outcome: *Harm to child:* Skin damage, pain or distress relating to gastrostomy site

Example 2

Patient Mickey gastrostomy was changed to a Mini Gastrostomy by nursing staff. Dietetic team were notified of change via voicemail received [date] p.m. Patient was sent home without correct extension set to use with new low - profile gastrostomy. Patient mother phoned [date -three days later] a.m to notify team that patient has been unable to receive supplemental enteral feed since change of gastrostomy button as has not had correct extension set.

Discussion at team meeting with CCN to take on the responsibility of finalising equipment before sent out to families. Further training provided on accurate note keeping. Team administrator not to lead on equipment decisions where clinical information / knowledge is required. CCN to contact relevant professionals when equipment changes are requested to gather accurate information. Issue with note keeper not naming key professional in the CCN notes. Training issue identified.

Care problem:

i) *Required equipment, medication and feeds not supplied at discharge:* Patient sent home without correct extension set to use with new low-profile gastrostomy

Contributory factor:

i) *Staff factors*: Equipment decisions made by team administrator who doesn't have the required clinical knowledge

Outcome: *Potential harm:* Child doesn't receive supplementary feed for three days
