# Supplementary material for: Paediatric enteral feeding at home: an analysis of patient safety incidents
Source: Arch Dis Child. 2019 Jun 14;104(12):1174–80. doi: 10.1136/archdischild-2019-317090 (PMC6900243; doi:10.1136/archdischild-2019-317090)
Supplement: Supplementary file 2 [file archdischild-2019-317090supp002.pdf]

Supplementary File 2: Description of the stages of analysis for coding the data.

**Step 1:** Familiarisation	Two authors (BP and RN) first familiarised themselves with the data by reading and rereading the reports and identifying and noting relevant themes.
**Step 2:** Coding the first 10% of incidents	The selected incidents were imported into NVivo, version 12. two researchers (BP and RN) initially coded 10% (n=35) of the incidents independently identifying whether the incident met the inclusion criteria and if it did, identifying i) the care management problems, ii) the contributory factors and iii) any stated outcome. The coding was compared, and any discrepancies resolved through discussion with the research team. Initial agreement was over 90%. There could be up to three care management problems and up to three contributory factors per incident. Outcomes were classified into 'clearly stated harm', 'potential harm' and 'no harm'.
**Step 3:** Coding the remaining incidents	The remaining incidents were split equally between the two researchers with each coding another 45% (n=156) of the incidents.
**Step 4:** Creating and revising the frameworks	The care management problems were grouped into sub-themes and themes through an iterative process. The two authors (BP and RN) developed the framework together, informed by existing frameworks. There are multiple other frameworks which have been developed for hospital care,[26,39] and one which has been designed for primary care.[24] These framework could not be applied directly to the narrow topic area of enteral feeding at home but they were adapted for use in this study. The contributory factors were also grouped into themes using existing frameworks as a model.[24-26]
**Step 5:** Final checks of the coding and the frameworks.	Incidents which were unclear were verified through discussion with the research team including author AL, a paediatric surgeon and author SH, a community paediatrician. A sample of 10% of the incidents were coded independently by the third author (AL), who has significant clinical expertise in surgical enteral feeding. Agreement was 100% for the outcomes and care problems and more than 95% for the contributory factors. A fifth author (CV) carried out a final check of the frameworks.
